# Notes on Myotomy and Tenotomy

**Published:** 1841-09-25

**Authors:** Reynell Coates


### ORIGINAL COMMUNICATION.

### NOTES ON MYOTOMY AND TENOTOMY.

BY REYNELL COATES, M. D.

To the Editors of the Medical Examiner.

Gentlemen,—In continuation of the subject of my last communication, let me ask of the friends of tenotomy in almost all cases of club-foot, what are the forces to be overcome in order to bring the member into a natural, or even into a useful position?

As there are many kinds of this deformity, and differences of degree in each, which are regarded as important by authorities, while, in fact, the principles on which the pathology and therapeutic treatment should be regulated are nearly identical in all the forms, it is best to select one only as the type of the affection, making incidental remarks upon the others as occasion may require. I have chosen varus in its most perfect state as the basis of the following remarks, both as the most common variety, and according to many, the most difficult of treatment by mechanical measures.

What, then, are the difficulties of greatest importance here? The most obvious, at a first glance, is the shortening of the extensors, and the adductors of the foot, which draw the heel upward until its longitudinal axis is nearly, or exactly parallel with that of the tibia, while, at the same time, they tilt the os calcis inward, until the inner side comes more or less nearly into proximity with the internal condyle, and the sole of the foot presents towards the opposite leg. Were this the only change effected by the morbid cause, we should have a kind of pes equinus, with the whole foot rotated inward until the upper articular surface of the os calcis would nearly approach the inner face of the astragalus, which very rarely changes its relative position, laterally, in varus. The difficulty could then be overcome with great facility, by simply dividing the tendons of the flexors and adductors, for the *only* cause of the deformity would be the action of these muscles. But this is, unfortunately, *not the only change.*

Experience proves that there is scarcely any alteration of the relative position of the three cuneiform tarsal bones, together with the cuboid and the bones of the metatarsus; or, in other words, there is little change in the form of the foot, anterior to the articulation of the cuboid with the os calcis and that of the cuneiform bones with the scaphoid bone. But, the cuboid is compelled to follow the motion of the os calcis, carrying with it the cuneiform bones, and also, the whole anterior portion of the foot, stretching the exterior or superior cubo-calcanean ligamentous fibres, and causing their permanent elongation. Meanwhile, the scaphoid bone necessarily partakes in the motions of the cuboid and cuneiform bones. It rotates upon its proper axis until its inner tuberosity presents upwards, and its outer surface downwards, perpendicularly, while the cuboid bone lies directly *underneath it*, if we speak of the foot in its natural position. Regarding the extention and tilting of the os calcis as the first of the elementary changes of position in varus, the rotation of the scaphoid and cuboid bones might be regarded as the second. But there is still another range of changes, even more important than these.

Every surgeon who is a mechanist, (as all ought to be so) well knows that the greater the adduction of the foot, when carried beyond its proper limits, while the astragalus remains stationary, the greater is the mechanical disadvantage under which the adducting muscles act—while, on the other hand, the more the plantar surface is turned towards the opposite limb, the more complete is the power of the adducting muscles and the long flexor of the toes to double the foot upon itself, along the middle joint of the tarsus already described.

From the moment that the os calcis begins to be rotated inwards, upon its axis, beyond the proper point, all these muscles collectively commence retracting by their tonicity, thus carrying the flexion of the anterior portion of the foot upon the os calcis and the astragalus to an unusual extent. The scaphoid sinks downward upon the head of the astragalus, leaving it painfully prominent upon the instep, while, in its completely adducted position, the flexing force being changed from the proper direction, slides it round upon the inner side of this bone. The os cuboides, also,—and for the same reason,—takes up a new position on the inner side of the os calcis, and the plantar surface of the fore part of the foot, instead of looking inward towards the opposite limb, as the under surface of the os calcis does, at least partially, looks directly backwards, and the toes extend directly towards the opposite ankle. It is this same excessive flexion in the new position of the parts, rather than a continued adduction, properly so called, that converts, what would have been a pes equinus with luxation of the calcis on the astragalus, into an extreme case of varus, with the metatarsal bone of the little toe resting on the soil.

To give a rude idea of the relative position of the bones in extreme varus to those who have not completely fixed in their minds the connections of all the tarsal and metatarsal bones, let us suppose the anterior portion of the foot to have been amputated between the os calcis and the astragalus, on the one hand, and the cuboides and scaphoides on the other. Then cutting the firm astragalo-calcanean interosseous ligament, subluxate the os calcis inward. Then take the amputated portion of the foot: turn it with the little toe downwards, and the plantar surface backwards, and place it in contact with the stump, so that the scaphoid bone should rest on the inside of the astragalus, and the cuboid on the inner and lower face of the anterior portion of the os calcis.

Rude as this sketch is, it will be found useful as a reference when I come to speak of the errors of indication in the construction of apparatus.

While in this condition, it is evident that the extremities of the metatarsal bones, next to their tarsal articulations, are brought much nearer than they should be to the heel, and this proximity is often still further increased on the inner side of the foot by a morbid flexion of the three cuneiform bones upon the os scaphoides. The immediate effect of this is a positive shortening of the distance between the origin and insertion, not only of the flexors and adductors of the whole foot, but of the short muscles of the toes also, together with the powerful fascia plantaris. And all these parts must necessarily undergo the several changes of function and structure observable in similar tissues under similar circumstances, after common luxations; and the laws that govern such changes are so well known in the present state of physiological knowledge, that they may be judged of *a priori*, without even having resort to observation: yet we shall find hereafter, that they have been viewed in a very one-sided manner by the exclusive advocates of the knife. What most interests us now is, the fact that the fascia plantaris,—in common with the muscles, after they have accommodated themselves to their new position as far as their tonic contractile power permits,—undergo an alteration in their very nutrition. They become permanently shortened, *and incapable of immediate extension*, at the same time that they are rendered weak for want of functional exercise.

Here, then, we have forces opposing the restitution of the tarsal bones to their proper relative position, which could not possibly be overcome solely by cutting the tendons of the tibial muscles, the triple flexor of the foot, and the long flexor of the toes. Suppose that these few sections have been made, and,—waving all new connexions between the parts, and the alterations of the form of the bones themselves,—what would be the position of the foot if the surgeon should attempt to restore it by the hand ?

All resistance to the flexion of the os calcis and its rotation outwards being removed by the knife, the posterior part of the sole of the foot would be readily directed downwards, and the axis of the os-calcis would be brought to a right angle with the leg. The proper relations between the articular surfaces of this bone and the astragalus being re-established, the cuboid bone would also return nearly to its place; but both this and the scaphoid would be flexed on the calcis and astragalus as far as the greatly relaxed ligaments of the instep would permit. The toes would be directed almost perpendicularly downward. The doubling of the middle of the foot would remain nearly unchanged. The exaggeration of the arch of the instep would be scarcely diminished, and any attempt to extend the anterior portion of the foot would be resisted by the habitually and permanently contracted ligaments of the under surface of that arch, by all the muscular fibres of the sole, and by the shortened fascia plantaris. Such are not *precisely* the appearances presented after the usual operation of tenotomy in valgus, but a considerable degree of this deformity is invariably present, increased and much modified by another that will be presently explained.

Now, the stretching of these contracted ligaments and muscular fibres requires either great force or a long time. It requires the very careful management of machinery; and a little want of mechanical tact may render that machinery quite as intolerable to the patient as any apparatus for the simple mechanical treatment, without tenotomy, is described to be by the enemies of that method. During the period of time in which the tenotomists are in the habit of employing mechanical extension, the new bond of union between the extremities of the tendons at the place of division continues to yield to extension. How long it may continue subject to such extension, may be made the subject of another note. This very extensibility of the newly deposited matter causes the mechanical force to act at a disadvantage in removing the undue flexion of the anterior part of the foot, and this disadvantage is exactly proportionate to the facility of extension. Let me explain. The fascia plantaris, and the muscles and ligaments of the sole of the foot are mainly connected at one extremity, with the os calcis.—When force is exerted after the division of the tendo Achilles, almost immediately after the operation, the os calcis occasionally flexes to a certain distance, (sometimes to its full extent,) very readily. The heel is then brought down with great facility; but, for this very reason, the flexion of the anterior upon the posterior portion of the foot, is scarcely affected at all; for the heel follows the traction of the shortened muscles and ligaments, leaving the exaggeration of the arch of the instep unaltered. If, on the contrary, the extension is delayed until the tendinous union remains firm, the force acts in extending the forepart of the foot, as well as in stretching the tendon; but its efficiency in effecting the former purpose is diminished by its expenditure, in part, upon the latter; and in order to accomplish the purpose, the operation of the force must be continued for a much longer time, and must be changed in its direction. As a matter of fact, the weight of *really well-conducted and dependable observations*, goes to show that this kind of deformity never has been entirely removed: not, as I believe, because it is positively incurable, but because the treatment is not wisely directed towards this end. In Delpech's case, twenty years of exercise had not wholly removed it.

But, say the tenotomists, this difficulty may be removed by dividing the fascia plantaris. This would alleviate, though it would not wholly remove the difficulty. In fact, it has not proved really and fully successful in practice, if we exclude, as every surgeon will, the reported cases in which the surgeon sweepingly asserts, that, in ten days, or three months, or six months, the patient walked as well as any body; *perhaps* adding, *merely incidentally*, in the next paragraph, that he continued to use Scarpa's, or some other peculiar shoe, *merely as a matter of precaution.* Such cases are utterly worthless, and ought to be rejected at once in all calculations of results.

The division of the fascia plantaris, were it not for certain after consequences that may possibly affect the question, would be advisable, on the plea of the difficulty of modifying dense ligaments by pressure or extension, were the causes of resistance to the return of the bones into their proper position entirely, or even mainly confined to those already mentioned: but this is far from being the case. The bones cannot assume the relative position described as characterizing complete varus, without extensive modifications of their form and their articulations.

The os calcis, in gliding towards the inner face of the astragalus, remodels the edge, and even the body of that bone, and its own inner edge undergoes reciprocal changes. The projecting process of the astragalus hanging downwards from the inner side of its posterior articulating surface is usually absorbed, and the corresponding edge of the anterior articulating surface is rounded off, while, in old cases, the body of the bone is reduced to an irregular wedge, with its edge directed inwards, and so thinned as to permit the angular upward projection of the inner edge of the os calcis to form strong ligamentous connections with the tibia; and, sometimes, when the internal condyle is not too far destroyed by absorption, it actually forms a new and complete capsular joint with that process.

Again; the tuberosity of the scaphoid bone is generally connected by new ligaments, and sometimes by a regular articulation with the malleolus. This bone is occasionally so thinned down at its lower and inner edges, that the length of that side of the foot is much diminished—a change that is rendered more remarkable by the diminution of nutrition in the whole leg and foot, but more especially observable on the inner side. In addition to all this, the astragalus is drawn into forced extension by the motion of the os calcis, and thus the whole foot is subluxated forwards. The posterior edge of the tibial trochlear cavity comes into contact with the os calcis, and new ligaments, and sometimes a new articulation is there formed between them.

So much for the changes in the bones, and the formation of new bonds of union between them. But what becomes of the old articulations in the meanwhile? The front and outside of the head of the astragalus has lost its articular cartilage. The capsular ligament has travelled round towards the inner and under surface of the body and head of the bone. The anterior portion of the pully of the ankle has also lost its cartilage, and the capsule has retreated to the middle of that surface, while the distance between the two malleoli is increased by absorption in front, and diminished by contraction in rear, until there is not space enough to receive the pulley, even were all other obstacles removed.

Now, varus of the most perfect form and of many years standing is cured, say the tenotomists, in from ten days to six months by mechanical treatment after division of the tendons, and, perhaps, the fascia plantaris! and the advocates of the purely mechanical treatment profess to do nearly or quite as much without resort to operation! Let me attack both parties alike. These gentlemen profess, in old and severe cases—What is it they profess? Thus much at least: They have caused the permanent elongation of a dozen new ligaments. They have restored the cartilages and capsular ligaments of several joints to their original position. They have destroyed by pressure or extension, three or four entire and well formed new articulations: and they have reconstructed the absorbed edges of the astragalus and os calcis, restoring the wedge-shaped body of the former to its original oblong form, all in from ten days to six months time! The purely mechanical therapeutists have done more! They have also elongated the muscles of the calf, the tibia and the sole of the foot, all completely adapted to their new position and modified in length and structure, sometimes so as to present more the appearance of common cellular tissue with its cells containing grains of a yellow fatty looking matter, (the inevitable result of long-continued muscular inactivity,) reconverting these organs to the condition of true and active muscles—all in from ten days to six months!

That this wonderful change may be accomplished—that it may sometimes be accomplished much more certainly without tenotomy than by its aid, the well known history of true luxations and the effects of exercise and rest most fully prove. But, *in from ten days to six months!* Truely this is asking credulity *beyond the faith of sailors!*

But "facts are facts," say the race of judges who smile at theory because they are too blind to see that theory (I do not mean *hypothesis*) is built exclusively on facts, and must be just as true as the facts on which it is founded. To this empirical school of observers I must say, that the results set forth in the reports of some of the boldest operators of either class, *are not strictly facts*, in the philosophical sense of the word. *Men who are too cautious to draw upon their imagination for their facts, may still be biased by their wishes!* The *cures*, in the bad cases, are not cures at the time of the reports. The very fact that they are so considered, probably prevents them from becoming so in a reasonable time! I have examined several cases of reported cures, where, indeed, the deformity has been astonishingly lessened, and the foot has been rendered useful to a considerable extent;—but in every severe case, whether treated with or without tenotomy, the exaggeration of the arch of the instep from contraction of the sole continued—the inner edge of the foot preserved its arcuated form from the diminution of the tarsal bones—the foot could not be flexed materially beyond the right angle—its subluxation forward was but partially removed, the pulley of the astragalus being decidedly prominent on the instep—and, although when the muscles were perfectly at rest the foot assumed the natural, partially adducted attitude of sleep, the moment the muscles were called into play, the tendency to turn preternaturally inwards was obviously betrayed—not so much, as it would seem, from excessive action of the adductors, as from the want of proper osseous support from the astragalus and os calcis.

But I am exceeding my proper limits, and must close for the present.

